# Insights into perceived listening difficulties post COVID-19 infection: no measurable hearing difficulty on clinical tests despite increased self-reported listening effort

**DOI:** 10.3389/fneur.2023.1172441

**Published:** 2023-05-18

**Authors:** Sara Alhanbali, Ana'am Alkharabsheh, Wafa'a Alanati, Khader Joudeh, Kevin J. Munro

**Affiliations:** ^1^Department of Hearing and Speech Sciences, School of Rehabilitation Sciences, The University of Jordan, Amman, Jordan; ^2^Manchester Centre for Audiology and Deafness, School of Health Sciences, University of Manchester, Manchester, United Kingdom; ^3^National Institute for Health and Care Research Manchester Biomedical Research Centre, Manchester University Hospitals National Health Service Foundation Trust, Manchester Academic Health Science Centre, Manchester, United Kingdom

**Keywords:** COVID-19, listening effort, fatigue, psychology, cognition, sub-clinical hearing loss

## Abstract

**Objective:**

The aim was to use a battery of clinic-based auditory assessment procedures to compare participants with and without self-reported hearing difficulties following a confirmed COVID-19 infection. A further aim was to compare the groups on self-reported measures of listening effort and fatigue.

**Methods:**

There were 25 participants in each group (age range 20–59 years, 80% females). Participants were recruited after a minimum of 4 weeks of testing positive. Hearing assessment involved tympanometry, acoustic reflex thresholds, pure-tone audiometry (PTA; 0.25–14 kHz), and distortion product otoacoustic emissions (DPOAEs; 0.5–10 kHz). Listening effort was assessed using the Arabic version of the Effort Assessment Scale (EAS-A) and fatigue was assessed using the Arabic version of the Fatigue Assessment Scale (FAS-A).

**Results:**

There was no difference between groups on any measure except for greater self-reported listening effort in the perceived hearing difficulty group (*p* = 0.01).

**Conclusion:**

The only difference between groups was self-reported listening effort. This could be due to a subclinical auditory deficit following COVID-19, increased listening effort due to the impact of COVID-19 on cognitive processes, or a psychosomatic response/health anxiety.

## Introduction

The World Health Organization (1) defines Coronavirus Disease (COVID-19) as an infectious disease caused by the SARS-CoV-2 virus (1). The majority of acute COVID-19 symptoms (such as fever, cough, and shortness of breath) resolve within 2–4 weeks. However, the UK National Institute of Health and Care Excellence [NICE; (2)] reported that COVID-19 can have long-term symptoms that persist from 4 to 12 weeks after the acute phase and referred to this as “ongoing symptomatic COVID-19”. NICE has also reported that some symptoms persist for more than 12 weeks and referred to this as “post-COVID-19 syndrome” (2). Symptoms of post-COVID-19 syndrome can include tinnitus, dizziness and otalagia (2).

Viral infections can cause hearing loss in some people. This is usually sensorineural but can vary in severity and be unilateral or bilateral (3). When considering the effect of COVID-19 on the audio-vestibular system, a systematic review by Almufarrij and Munro (4), reported the lack of: (i) studies that employed an objective, comprehensive assessment of auditory function in COVID-19 patients, and (ii) controls. Since this review, the number of COVID-19 studies with controls has increased (5–7) but there is a lack of agreement on the impact on the auditory system.

Recent studies that employed structured methodological approaches reported absent effect of COVID-19 on hearing. For example, Taitelbaum-Swead et al. (8) reported no difference in hearing sensitivity pre and post-COVID-19 infection (after controlling for age). When considering patients with severe COVID-19 symptoms, Visram et al. (in press), also did not identify a difference in auditory function between 57 hospitalized COVID-19 patients and 40 matched non-COVID hospitalized controls when participants' hearing function was thoroughly investigated using lab-based behavioral and physiological measures.

Perceived listening difficulties can stem from deficits that cannot be always identified using standard audiometric tools. For example, the experience of listening effort and fatigue does not often correlate with the amount of hearing disability (9, 10).

Despite the absence of auditory effects in recent studies, there are people who report hearing-related symptoms. There have been reports suggesting that the perceived difficulties may have a psychological basis or reflect the limitation of relying on self-report measures (11). In order to investigate this further, the first aim of our study was to use a battery of clinic-based auditory assessment procedures to compare participants with and without self-reported hearing difficulties following a confirmed COVID-19 infection. Because of the reports of post-COVID-19 fatigue (12), a further aim was to compare the groups on self-reported measures of listening effort and fatigue. The results of the auditory assessment procedures will confirm the presence of a hearing deficit. However, absent measurable difference in hearing sensitivity with a difference in self-report measures could suggest the presence of a subclinical auditory deficit, an impact of COVID-19 on cognitive processes, or a psychosomatic response/health anxiety. The outcomes of this study will be a first step toward explaining perceived listening difficulties in some COVID-19 patients.

## Materials and methods

### Participants

A minimum sample size of 64 participants per group was estimated to provide 80% statistical power to detect a clinically significant difference with a medium sized effect; *r* = 0.3 (13), between the groups (α = 0.05), based on an independent *t*-test. A total of 50 participants took part in this study. Achieving the target sample size was limited by difficulties in identifying enough participants who reported hearing-related symptoms post-COVID-19 infection. The traveling restrictions imposed during the time period of the data collection (to control the spread of the virus) have also limited the number of participants recruited. There were 25 participants in the group of participants who reported hearing-related symptoms after COVID-19 infection [perceived difficulty (PD)] and 25 sex- and age-matched (within 24 months) participants in the group of participants who did not report an effect for COVID-19 on hearing (no-PD). Participants' age range was 20–59 (mean = 30). Eighty percent of the participants were female.

The announcement for recruiting participants in the PD group stated that potential participants need to have noticed an effect of COVID-19 on their hearing abilities or have started experiencing tinnitus after COVID-19 infection. Potential participants contacted researcher SA by telephone. Participants were asked to describe their hearing related symptoms post-COVID-19 infection. Those who reported deterioration in hearing sensitivity with or without tinnitus were invited to take part in the study. Perceived hearing difficulty with or without tinnitus was reported by 24 participants in the PD group. A single participant reported experiencing tinnitus only without any hearing difficulty. Fourteen participants reported PD with tinnitus while the rest reported PD only. Participants in the PD group were approached through hospitals and ENT clinics and through announcements and videos posted on social media platforms. For participants in no-PD group, the announcements stated that researchers were looking for participants born on a specific year (to match those in the PD group) and who were infected with COVID-19 at least 4 weeks ago to complete a series of hearing tests. Participants in the no-PD group were approached through announcements and videos posted on social media platforms. There was no mention of the hypothesis that COVID-19 might negatively impact hearing sensitivity in any of the studys' advertisements. There was about 8 months gap between the recruitment of the participants in each of the groups which decreases the possibility that participants in the no-PD group have seen the adverts for the PD group. The advertisements stated that there would be a financial reward for taking part in the study and that the duration for the whole testing will be 45–60 min.

All participants had confirmed COVID-19 at least 4 weeks before taking part in the study. The duration ranged from 4 weeks to 12 months with a mean of around 6 months for each of the groups. There was only one participant in the no-PD group who was infected 24 months before taking part in the study. COVID-19 infection was confirmed by the reverse transcriptase polymerase chain reaction (RT PCR). All participants were symptomatic during the infection. Participants were asked to rate the severity of the symptoms they experienced on a scale from 0 to 10 with 10 indicating most severe symptoms. Responses ranged from 2 to 10 with an average of 6.5. Eighty percent of the participants also reported experiencing fever during the infection. None of the participants were hospitalized but two of the participants reported requiring home treatment (e.g., ventilators) and monitoring due to the severity of the symptoms. Participants were asked about any medication that they have consumed during the infection and the majority of them reported taking paracetamol and multivitamins only.

Before taking part in the study, participants in both groups were asked about any family history of hearing loss and any history of noise exposure. In the OSHA pocket guide for Occupational Safety and Health Administration (14), the “Noise Indicator Foot Rule” states that having to speak loudly in order to be heard by someone who is two to three feet away (around 1 m) suggest that the noise level is above 85 dBA which is damaging to the auditory system. Therefore, noise exposure was explained to the participants as spending about 8 h every day in a noisy environment where they need to shout in order to communicate with people who are 1 m away of them. This was also based on the recommendations of the National Institute for Occupational Safety and Health [(15), NIOSH] which suggested that hearing damage is likely to occur when noise exposure is at or above 85 dBA averaged over 8 working hours. It should be noted that acoustic trauma can occur as a result exposure to sounds that are less intense than 85 dBA for prolonged periods of time. For example, NIOSH stated that exposure to a sound level of 81 dBA can result in damage to the auditory system if the duration of exposure exceeded 20 h daily. Here, results of DPOAEs and pure tone audiometry suggest that hearing damage as a result of noise exposure is unlikely in the participants tested (see results section below). Participants who reported family history of hearing loss or history of noise exposure were excluded from the study. Two participants reported noise exposure at the work environment and were therefore excluded. Participants in the no-PD group were asked if they believe they have a hearing difficulty to check if their decision to take part in the study was influenced by the possibility of having a hearing problem that they wanted to get checked. However, all participants in the no-PD denied being aware of any hearing difficulty. The study was reviewed and approved by the Deanship of Scientific Research at the University of Jordan (2020-22/IRB).

### Procedures

After obtaining written consent from each participant, otoscopic examination and tympanometry were first performed. The hearing tests were conducted by three audiologists, all authors, who work at the University of Jordan. An Otometrics Madsen Otoflex 100 tympanometer was used for the assessment of the middle ear function. Tympanometry was conducted using a 226 Hz probe tone. Acoustic reflex was performed using a 226 Hz probe tone at the frequencies 0.5, 1, and 2 kHz. ARTs at frequencies above 2 kHz tend to be elevated or absent even in young individuals with normal hearing (16) and therefore were not tested. A manual procedure in which the audiologist determines the acoustic reflex threshold (ART) at each frequency was followed. A reflex threshold was defined as the lowest intensity that elicits a minimum of 0.02 mmho change in compliance.

Pure tone Audiometry (PTA) was used to assess participants' hearing thresholds in the frequency range 0.25–14 kHz using an Interacoustics AC40 Clinical Audiometer. Both octave and inter-octave frequencies were tested. Air conduction hearing thresholds were assessed for all participants. Bone-conduction hearing thresholds were only assessed if the air conduction thresholds were 20 dB HL or poorer. A TDH-39 headphones were used for the assessment of hearing in the frequency range 0.25–8 kHz and a circum-aural (HDA200) for the assessment in the frequency range 10–14 kHz. PTA test was performed in a sound treated room.

Distortion Product Otoacoustic Emissions (DPOAEs) were performed using Otometrics Madsen Capella device at the frequencies 0.5, 1, 2, 4, 6, 8, and 10 kHz. Each DP was elicited using two Primary frequencies (F1 and F2) that are related by the equation (2F1 – F2 = DP). F1–F2 ratio was 1.22. The intensities of F1 and F2 were 65 dB SPL and 55 dB SPL, respectively. A response was considered present if the difference between the signal and the noise floor (SNR) was equal to or more than 6 dB.

Participants completed the Arabic versions of the Effort Assessment Scale; EAS-A and the Fatigue Assessment Scale; FAS-A (17). The EAS-A (Supplementary material 1) consists of 6 questions that assess the experience of listening effort in everyday life, e.g., “*Do you have to put in a lot of effort to hear what is being said in conversation with others*?” Responses are provided on a visual analog scale ranging from 0 to 10 with 10 indicating more effort. An average score was calculated for the entire EAS-A items. Average scores were then converted into percentages. The FAS-A (Supplementary material 2) is a generic scale which includes nine items that assess both the physical and mental fatigue, e.g., “*Mentally, I feel exhausted*”. Response options range from “never” to “always” with never equivalent to 1 and always equivalent to 5. Opposite scoring is used for items 3 and 9. An average score was calculated for the entire FAS-A items. Average scores were then converted into percentages.

### Analysis

Normality of the data was checked using Shapiro-Wilk and Kolmogorov-Smirnov tests. Results suggested that the hearing threshold, EAS-A and FAS-A data were not normally distributed (*p* < 0.05). Therefore, Mann–Whitney *U*-test was used to identify the presence of a significant difference between groups. For the remaining data, independent sample *t*-test was used when comparing groups.

## Results

### Otoscopy and tympanometry

Results of otoscopy and tympanometry suggested normal outer and middle ear function for all participants.

### Acoustic reflex thresholds

Kruskal-Wallis test was first performed to identify the presence of a significant difference between the ARTs of the right and the left ears at the frequencies 0.5, 1, and 2 kHz. As suggested by the figure in Supplementary material 3, results of the statistical analysis revealed the absence of a significant difference between the ARTs of the right and the left ears. Therefore, ARTs for the different frequencies were averaged across the right and the left ears.

**Figure 1:**
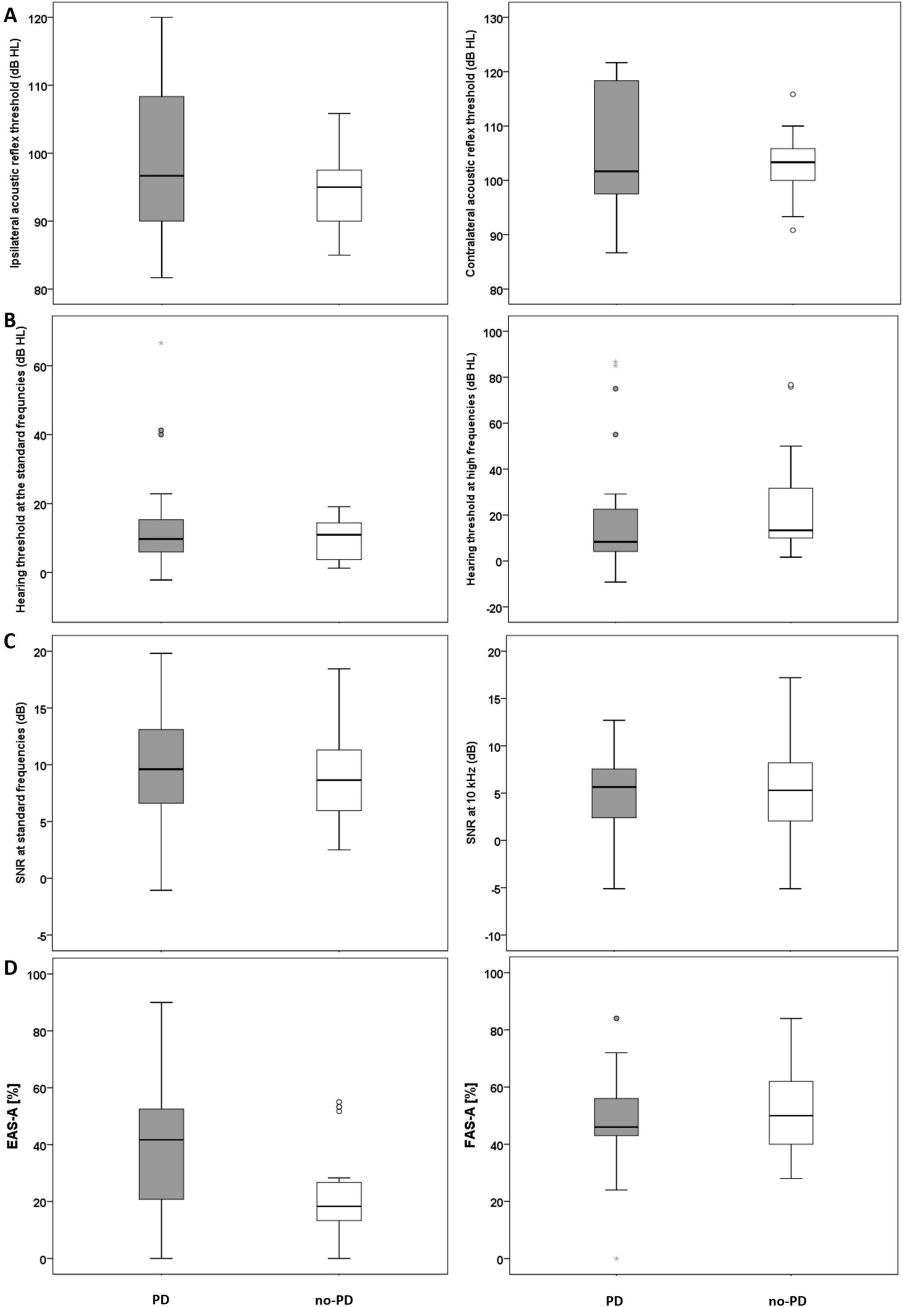
**(A)** Mean ART data (ipsilateral, left panel; contralateral, right panel) for the PD and the no-PD groups. **(B)** Mean hearing threshold across the standard frequency range (left panel) and at the high frequency range (right panel) for the PD and the no-PD groups. **(C)** Mean DPOAEs SNRs across the frequencies 0.5–8 kHz (left panel) and DPOAE SNR at 10 kHz (right panel) for the PD and the no-PD groups. **(D)** Self-reported listening effort (Left panel) and self-reported fatigue (right panel) for the PD and the no-PD groups. The solid horizontal line in the middle of each box plot represents the median score. Each box represents the upper and the lower quartiles of the data (the middle 50%). The distance between the upper quartile and the top whisker is the range of the top 25% scores. The distance between the lower quartile and the bottom whisker is the range of the bottom 25% scores. Circles represent outliers that are more than 1.5 times the interquartile range (the range between the upper and the lower quartile). Stars represent outliers that are more than 3 times the interquartile range.

Mean ipsilateral ART (frequencies 0.5–2 kHz) was 98.1 (SD: 2.1) for the PD group and 94.5 (SD: 5.2) for the no-PD group. Mean contralateral ART was 105.4 (SD: 11.1) for the PD group and 103.1 (SD: 5.4) for the no-PD group. Results of independent *t*-test revealed no significant between the two groups in mean ipsilateral ART (frequencies 0.5–2 kHz) *t*_(48)_ = 1.6, *p* > 0.05 (Figure 1A; left panel) and or in mean contralateral ART (frequencies 0.5–2 kHz) *t*_(48)_ = 0.99, *p* > 0.05 (Figure 1A; right panel).

### Puretone audiometry

Kruskal-Wallis test was first performed to identify the presence of a significant difference between the hearing thresholds of the right and the left ears at the different frequencies tested (0.25–14 kHz). As suggested by the figure in Supplementary material 4, results of the statistical analysis revealed the absence of a significant difference between the thresholds of the right and the left ears. Therefore, pure-tone thresholds for the different frequencies were averaged across the right and the left ears.

Median hearing threshold across the standard frequency range (0.25–8 kHz) was 10 dB HL (IQR: 9) for the PD group and 11 dB HL (IQR: 11) for the no-PD group. For extended high frequencies 10–14 kHz), median hearing threshold for the PD group was 8 dB HL (IQR 18) and 13 dB HL (IQR: 25) for the no-PD group. Results of Mann-Whitney U test revealed no significant difference between the two groups at standard frequencies *U* = 305.5, *p* = 0.89 (Figure 1B; left panel) or at extended high frequencies *U* = 250.5, *p* = 0.23 (Figure 1B; right panel).

### Distortion product otoacoustic emission

Kruskal-Wallis test was first performed to identify the presence of a significant difference between the SNRs of the right and the left ears at the different frequencies tested (0.5–10 kHz). As suggested by the figure in Supplementary material 5, results of the statistical analysis revealed the absence of a significant difference between the right and the left ears. Therefore, the SNRs for the different frequencies were averaged across the right and the left ears.

Mean DPOAEs SNR at the standard frequencies (0.5–8 kHz) was 9.7 (SD: 4.3) for the PD group and 8.9 (SD: 4) for the no-PD group. Mean DPOAEs SNR at 10 kHz was 4.8 (SD: 5) for the PD group and 4.7 (SD: 5) for the no-PD group. Results of independent *t*-test revealed no significant between the two groups at standard frequencies (0.5–8 kHz) *t*_(48)_ = 0.81, *p* > 0.05 (Figure 1C; left panel) or at 10 kHz *t*_(48)_ = 0.24, *p* > 0.05 (Figure 1C; right panel).

### Self-reported listening effort

Median EAS-A score was 41.7% (IQR: 35) for the PD group and 18.3% dB HL (IQR: 15) for the no-PD group. Figure 1D (left panel) suggests that participants in the PD group reported increased listening effort compared to the no-PD group. Results of the statistical analysis confirmed that the difference between the groups is significant *U* = 168, *p* = 0.01.

### Self-reported fatigue

Mean FAS-A score was 48.1% (SD: 17) for the PD group and 52.8% (SD: 15.9) for the no-PD group. Figure 1D (right panel) suggests that participants in the PD group and participants in the no-PD group report similar levels of fatigue. Results of the statistical analysis revealed no significant difference between the groups *t*_(44)_ = −0.97, *p* > 0.05.

## Discussion

In this study, we performed a detailed investigation of auditory function in two groups of participants, one of which reported hearing difficulty after contracting COVID-19. Methods of hearing assessment included a series of objective tests that are used in clinical settings. We also investigated self-reported listening effort and fatigue. The only difference identified between the groups was in self-reported listening effort.

EAS-A scores in the no-PD group were comparable to those reported by the control group in Alhanbali et al. (18) who did not have a hearing problem (around 20%). However, median EAS-A scores of the PD group were around 42%, which is higher than the scores of the control group in Alhanbali et al. but still lower than the scores of the groups with hearing impairment (around 60%) in the same study. Perceived listening effort is more likely to correlate with the perceived hearing disability rather than hearing sensitivity (9, 10). That is, it is possible that some underlying deficits contribute to individuals' experience of listening effort that cannot be necessarily identified using the standard hearing assessment tools. It is important to note that absent difference in hearing sensitivity could be a result of the small sample size. However, factors that could contribute to increasing the experience of listening effort despite normal auditory function include: (i) a subclinical auditory deficit following COVID-19, (ii) the impact of COVID-19 on cognitive processes involved in listening, or (iii) a psychosomatic response/health anxiety.

### Potential causes of increased self-reported listening effort

#### Subclinical auditory deficit

The difference in self-reported listening effort between the groups could be a result of an underlying sub-clinical hearing loss such as hidden hearing loss. However, it is important to be cautious about this interpretation given that evidence so far suggests absent effect for COVID-19 on the neural function of the auditory system. Visram et al. (in press) and Dror et al. (19) did not identify a difference between recovered COVID-19 patients and controls in the outcomes of auditory brainstem response and acoustic reflex tests. More specifically, Visram et al. reported absent difference in peak-to-trough amplitudes for wave I, and intervals for wave I to wave V peaks I ABR amplitude between recovered COVID-19 patients and controls.

#### Potential effect of COVID-19 on cognition

The difference in listening effort could be a result of an effect of COVID-19 on cognitive functions involved in listening. Examples include attention, memory, and speed of processing which are known to influence individuals' experience of listening effort (20). Listening is a complex mechanism that does not solely depend on hearing sensitivity. There are reports suggesting that COVID-19 may have a negative impact on cognition. Executive function was found to be particularly affected by COVID-19 and to a lesser extent, memory (21).

Cognitive dysfunction can result from the neurological sequela of COVID-19. Causes of neurological dysfunction in COVID-19 patients include hypoxia associated with respiratory distress, prothrombotic state, peripheral inflammatory response, and direct viral invasion. The psychological consequences of COVID-19 such as stress, anxiety, and depression can also negatively impact cognition in COVID-19 patients. Increased prevalence of cognitive impairment has been identified amongst hospitalized COVID-19 patients compared to patients with mild symptoms (21).

It is important to consider that mixed finding have been reported across the studies that investigated the effect of COVID-19 on cognition. This is due to the variability across the studies in: (i) the inclusion criteria, (ii) time and methodology of assessment, and (iii) whether a control group was included as many studies did not include a control group (21). In a recent systematic review by Ceban et al. (12), the authors also reported that around fifth of the participants recruited in the 43 studies reviewed experienced persistent cognitive impairment 12 weeks after recovering from COVID-19. However, the authors highlighted the importance of interpreting the results with caution due to the presence of a number of limitations in the studies reviewed. For example, the majority of the studies reviewed were observational and causality could not be established. Additionally, a large number of studies failed to establish whether the impairments identified were present before COVID-19 infection. Future research should therefore consider assessing the cognitive function of individuals who report hearing related symptoms post-COVID-19 infection.

Given the effect that COVID-19 can have on cognition, listening difficulty due to Auditory Processing Disorder (APD) in some patients should not be overlooked. COVID-19 may impact cognitive functions such as auditory memory and the executive function which may be affected in the case of APD. Therefore, the possibility of APD post-COVID-19 infection should be further investigated using the recommended testing methods for this purpose.

#### Psychosomatic response/health anxiety

The difference between the groups in self-reported listening effort could potentially be attributed to psychological factors. In a recent study by Saunders et al. (11), the authors reported significantly increased self-reported hearing loss amongst the groups with confirmed and probable COVD-19 compared to a group of participants who were not infected with COVID-19. Around 60% of the participants with confirmed COVID-19 infection reported experiencing a symptom with no established association with COVID-19 (toothache) with a considerable overlap in the patients who reported that COVID-19 is the cause of their symptoms. The overlap in the symptoms reported was interpreted as possible nocebo effect which is defined as “new or worsening symptoms that develop in response to negative health-related information, beliefs, and/or experiences” (22). Symptoms with probable or no established association with the virus were mostly reported by participants with probable COVID-19. This was considered an indication of increased levels of health anxiety which might have led to somatization of the symptoms. Collectively, results of Saunders et al. suggest that self-report data need to be interpreted with caution given its increased susceptibility to bias and inaccuracy.

One thing to note is that no difference in self-reported fatigue was identified between the groups (see section below). This finding possibly rules out the assumption that the difference between the groups in self-reported listening effort is an indication of psychosomatic response/health anxiety in the PD group. In other words, a psychosomatic response/health anxiety in the PD group will likely result in a significant difference between the groups in all self-report measures. However, it is important to be cautious about this interpretation given the small sample size and the fact that the FAS-A is a generic scale of fatigue as will be discussed below.

### Self-reported fatigue

Self-reported fatigue was assessed using the FAS-A which is a generic scale that assesses both physical and mental fatigue. No difference was identified in self-reported fatigue between the groups. This finding is not surprising given that fatigue is one of the most commonly reported long COVID-19 symptoms (12) and the fact that the FAS-A is a generic scale of fatigue. In fact, raw fatigue scores for each of the groups (around 24 for the PD group and 26 for the no-PD group) are comparable to those reported in other studies that investigated long-term fatigue post-COVID-19 infection. For example, Serafini et al. (23) reported that mean raw FAS score was around 20 across a group of COVID-19 patients a few months after recovery. Visram et al. (in press) also reported a mean FAS score of 25.1 across recovered COVID-19 patients. Raw fatigue scores in the PD and no-PD groups are above the cut-off score of 22 which suggests significant levels of fatigue (24). Scores above 22 were commonly identified among groups of patients with chronic health conditions such as in patients with sarcoidosis (24) and post-stroke patients (25). Considering that the mean age of the participants in this study was 30 years and that most of them were approached from the general population and not from a particular site, we have no reason to believe that conditions other than COVID-19 are responsible for the increased self-reported fatigue. However, it is important that future research confirm this by ruling out the presence of conditions that contribute to the long-term experience of fatigue such as chronic illnesses, sleeping and eating disorders, and mental health conditions (26).

## Limitations and future directions

Future research should investigate possible causes for the significant difference in self-reported effort between the groups despite the similarities in the outcomes of the clinic-based auditory assessment procedures. This can be achieved by: (i) assessing the cognitive abilities of the participants, and (ii) investigating listening effort and fatigue using objective methods to overcome the limitation of the lack of reliability of self-report measures. Future research should also investigate fatigue post-COVID-19 more systematically by trying rule out the presence of confounding factors that contribute to the experience of fatigue.

## Conclusions

Increased self-reported listening effort was identified in participants who reported increased hearing difficulty after COVID-19 infection compared to participants who did not report an effect of COVID-19 on hearing. However, no difference in the results of multiple clinical hearing assessment tools was identified between the groups. The difference in listening effort might be a result of a subclinical auditory deficit following COVID-19, increased listening effort due to the impact of COVID-19 on cognitive processes, or a psychosomatic response/health anxiety. However, future research needs to implement more objective methods to investigate the underlying causes of the increased self-reported effort.

## Data availability statement

The raw data supporting the conclusions of this article will be made available by the authors, without undue reservation.

## Ethics statement

The studies involving human participants were reviewed and approved by the University of Jordan. The patients/participants provided their written informed consent to participate in this study.

## Author contributions

SA: study design, data acquisition, analyses, and writing up the manuscript. KM: study design and writing and critically revising drafts of the manuscript. AA, WA, and KJ: data acquisition. All authors contributed to the article and approved the submitted version.
