# Supplementary material for: Insights into perceived listening difficulties post COVID-19 infection: no measurable hearing difficulty on clinical tests despite increased self-reported listening effort
Source: Front Neurol. 2023 May 18;14:1172441. doi: 10.3389/fneur.2023.1172441 (PMC10233052; doi:10.3389/fneur.2023.1172441)
Supplement: Supplementary file 1 [file Data_Sheet_1.DOCX]

**Supplement 1. EAS-A items**

| Arabic | English |
| --- | --- |
| هل تضطر إلى بذل جهد كبير لسماع ما يقوله الآخرون أثناء إجراء حديث؟ | **Do you have to put in a lot of effort to hear what is being said in conversation with others?** |
| ما مقدار التركيز الذي تضطر إلى بذله عند الاستماع إلى أحد؟ | **How much do you have to concentrate when listening to someone?** |
| ما مدى سهولة تجاهلك للأصوات المحيطة عند الاستماع لشيء محدد؟ | **How easily can you ignore other sounds when trying to listen to something?** |
| هل تضطر إلى بذل جهد كبير لمتابعة نقاش في الصف الدراسي أو اجتماع أو محاضرة؟ | **Do you have to put in a lot of effort to follow discussion in a class, a meeting, or a lecture?** |
| هل تضطر إلى بذل جهد كبير لمتابعة حديث وسط الضوضاء (مثل مطعم أو جَمعَة عائلية)؟ | **Do you have to put in a lot of effort to follow the conversation in a noisy environment (e.g., in a restaurant, at family gatherings)?** |
| هل تضطر إلى بذل جهد كبير أثناء التحدث على الهاتف؟ | **Do you have to put in a lot of effort to listen on the telephone?** |
