# Supplementary material for: Insights into perceived listening difficulties post COVID-19 infection: no measurable hearing difficulty on clinical tests despite increased self-reported listening effort
Source: Front Neurol. 2023 May 18;14:1172441. doi: 10.3389/fneur.2023.1172441 (PMC10233052; doi:10.3389/fneur.2023.1172441)
Supplement: Supplementary file 2 [file Data_Sheet_2.DOCX]

**Supplement 2. FAS-A items**

| Arabic | English |
| --- | --- |
| يزعجني الإعياء (التعب الشديد) | **I am bothered by fatigue** |
| أتعب بسرعة | **I get tired very quickly** |
| لا أتمتع بالطاقة اللازمة للقيام بمتطلبات حياتي | **I have enough energy for everyday life** |
| أشعر بالإرهاق الجسدي | **Physically, I feel exhausted** |
| لدي صعوبة في البدء بعمل مهام حياتية | **I have problems starting things** |
| أستصعب التفكير بذهن صافٍ | **I have problems thinking clearly** |
| لا رغبة لدي للقيام بشيء | **I have no desire to do anything** |
| أشعر بالإرهاق الذهني | **Mentally, I feel exhausted** |
| ليس بإمكاني التركيز بشكل جيد عند القيام بأمر معين | **When I am doing something, I can concentrate quite well** |
