# Supplementary material for: Insights into perceived listening difficulties post COVID-19 infection: no measurable hearing difficulty on clinical tests despite increased self-reported listening effort
Source: Front Neurol. 2023 May 18;14:1172441. doi: 10.3389/fneur.2023.1172441 (PMC10233052; doi:10.3389/fneur.2023.1172441)

**Supplement 4.** The hearing thresholds of the right and the left ears at each frequency tested using pure-tone audiometry.


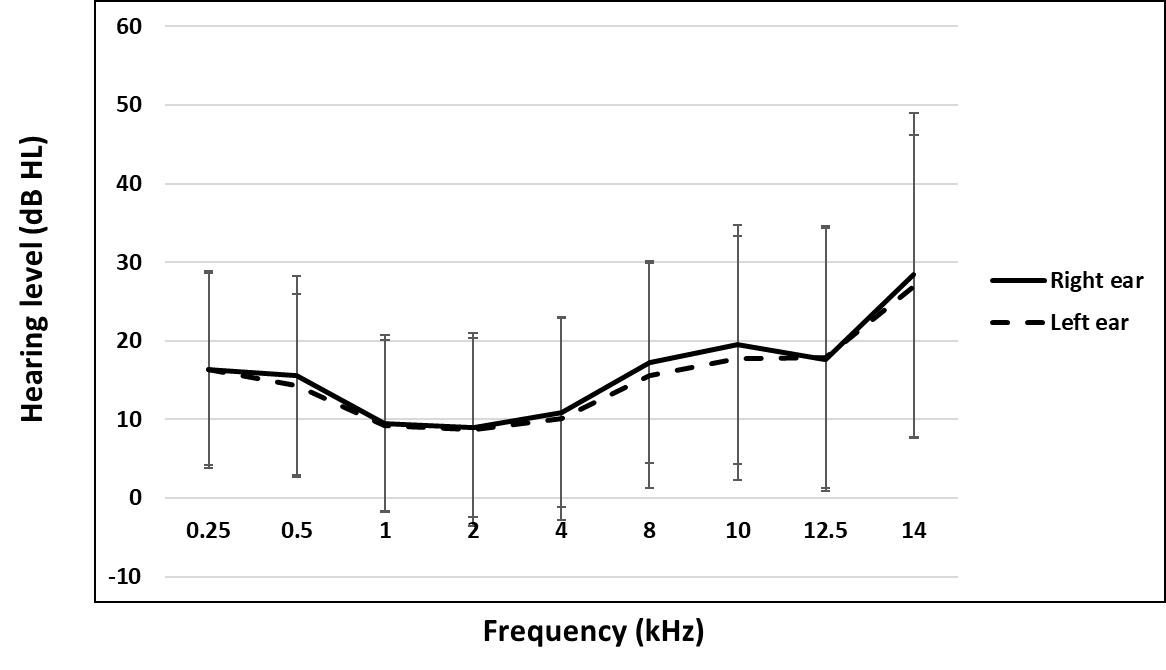

Supplement: Supplementary file 4 [file Data_Sheet_4.DOCX]
